# Assessment of the Impact of Anti-nuclear Antibody (ANA) Titer and Pattern on Anti-extractable Nuclear Antigen (ENA) Positivity: Experience at Cheikh Khalifa Hospital

**DOI:** 10.7759/cureus.85723

**Published:** 2025-06-10

**Authors:** Hamza Ouazzani, Hajar Fadili, Halima Filali, Abdelhamid Zrara, Youssef Bamou, Jalila El Bakkouri

**Affiliations:** 1 Biology, Cheikh Khalifa Ibn Zayed Hospital, Mohammed VI University of Health Sciences (UM6SS), Casablanca, MAR

**Keywords:** anti-ena, antinuclear antibodies, autoimmune diseases, diagnostic biomarkers, immunofluorescence

## Abstract

This retrospective study examined the relationship between anti-nuclear antibody (ANA) titers, immunofluorescence (IIF) patterns, demographic variables, and anti-extractable nuclear antigen (ENA) positivity in a cohort of 538 patients at Cheikh Khalifa Hospital. Among them, 409 of 538 patients (76%) were women, and 129 of 538 (24%) were men. ANA and 15 ENA specificities were assessed using indirect IIF on Hep-2/monkey liver substrates and immunoblot analysis with the ANA Profile 3 Plus DFS-70 kit (EUROIMMUN Medizinische Labordiagnostika AG, Lübeck, Germany). ANA staining patterns were classified based on ICAP guidelines as speckled (S-ANA), homogeneous (H-ANA), cytoplasmic (C-ANA), and nucleolar (N-ANA). Certain patterns are known to be associated with specific antibodies or clinical conditions; for example, S-ANA may suggest anti-Sm antibodies in systemic lupus erythematosus, while C-ANA is often linked to anti-mitochondrial or anti-Jo-1 antibodies, commonly seen in primary biliary cholangitis or antisynthetase syndrome, respectively. Chi-square analyses were used to identify key factors predictive of ENA positivity.

ANA was detected in 326 of 538 patients (60%). Among those who were positive, most were S-ANA, observed in 161 of 326 patients (49%). The second most common pattern was H-ANA, found in 49 of 326 (15%). Anti-ENA antibodies were identified in 268 of 538 patients (50%), with Ro-52, SS-A, and Pm-Scl100 being the most commonly detected specificities. Moderate and higher ANA titers (≥1:320) showed a strong association with ENA positivity. Our analyses indicate that high ANA titers and specific patterns, particularly S-ANA and H-ANA, are associated with ENA positivity and may aid in the interpretation of ENA tests. We also found that C-ANA didn’t show any predictive value for ENA positivity. In contrast, N-ANA did have some predictive value but less unequivocal relationships with some ENA antibodies. The study noted that more women and patients aged 40-60 showed positive anti-ENA results; however, these observations may be affected by the overrepresentation of these groups.

Furthermore, 47 of 538 patients (9%) who tested negative for ANA were still found to be anti-ENA positive, highlighting discrepancies that can occur between detection methods. These findings underscore the importance of integrating ANA profiles with the patient’s clinical context, including their medical history and examination findings, to enhance diagnostic precision. Moving forward, further studies with standardized assay procedures and prospective clinical follow-up would help refine the interpretation of serological results.

## Introduction

Anti-nuclear antibodies (ANA) are autoantibodies directed against various cellular components, including nuclear, mitotic, and cytoplasmic elements. Their detection is generally indicated in patients with signs suggestive of connective tissue disease or systemic autoimmune disease. ANA profiles are strongly associated with autoimmune conditions, such as systemic lupus and scleroderma, frequently serving as early warning signs. However, positive ANA results aren't unique to autoimmunity; we regularly detect low-titer ANA in healthy controls and patients battling infections or unrelated conditions. Typically, higher ANA titers are associated with a greater likelihood of true autoimmune pathology, whereas very low ANA titers (1:40 or 1:80) are often nonspecific. Previous surveys have shown that only a small percentage of healthy people (around 5%) have ANA titers as high as 1:160 [1,2].

They are most commonly detected by indirect immunofluorescence (IIF) on Hep-2 cells, a human cell line derived from a squamous cell carcinoma of the larynx, which is chosen due to its large nuclei and high mitotic rate, allowing for the screening of a wide range of autoantibodies. However, it does not precisely identify specific antigenic targets [3].

Therefore, following a positive ANA-IIF result, anti-extractable nuclear antigen (ENA) antibodies are often detected using solid-phase techniques such as ELISA, immunodot, or the Luminex method, allowing for a more precise identification of targeted antigens. Some patterns of ANA staining are associated with certain ENA antibodies or diseases; for instance, a speckled pattern might suggest anti-Sm in lupus, or C-ANA patterns are often associated with antibodies targeting cytoplasmic antigens, such as anti-mitochondrial or anti-Jo-1 antibodies, which are relevant in primary biliary cholangitis or antisynthetase syndrome, respectively. Whether the ANA titer level or IIF pattern can predict subsequent ENA positivity is an important question for optimizing diagnostic algorithms [4].

This study aimed to assess the association between patient demographics (age, sex), ANA titers, and IIF patterns with ENA antibody detection by immunoblot to evaluate their predictive value and support more targeted ENA testing. This study was conducted at Cheikh Khalifa Hospital, a tertiary care center in Morocco that serves a diverse patient population, providing a relevant and representative setting for evaluating ANA and ENA profiles in real-world clinical practice.

## Materials and methods

Study design*​​​​​​*

We performed a retrospective, observational study in the immunology laboratory of Cheikh Khalifa Hospital, reviewing all ANA and ENA tests conducted from January 1, 2023, to January 31, 2024.

Study population and sample size

During the study period, we included 538 consecutive patients referred for ANA and ENA testing. Exclusion criteria comprised hemolyzed or insufficient serum samples, as well as patients with a confirmed diagnosis of autoimmune disease who were already receiving immunosuppressive or immunomodulatory therapy. The latter exclusion aimed to minimize the confounding effects of treatments on autoantibody titers and profiles. The final cohort consisted of 538 patients, including 409 females (76.0%) and 129 males (24.0%), with a mean age of 47.7 years (range: 0-90 years). The age distribution was stratified as follows: 38 patients (7%) were aged 0-20 years, 161 (30%) were aged 20-40 years, 187 (35%) were aged 41-60 years, and 152 (28%) were over 60 years.

Laboratory procedures

ANA screening was performed at an initial dilution of 1:160 using Hep-2/monkey liver slides and FITC-conjugated anti-IgG (EUROIMMUN Medizinische Labordiagnostika AG, Lübeck, Germany). Fluorescence patterns were recorded and classified following the International Consensus on ANA Patterns (ICAP) guidelines, including homogeneous (H-ANA), speckled (S-ANA), cytoplasmic (C-ANA), nucleolar (N-ANA), centromeric (CE-ANA), and mixed, as well as end-point titers (1:160, 1:320, 1:640, ≥1:1 280) [5].

We considered a titer of 1:160 as the screening cutoff for positivity. Additionally, for analysis, we defined high-titer ANA as ≥1:1280, moderate titer as 1:320 or 1:640, and low titer as 1:160. All ANA IIF slides were interpreted by qualified specialists in clinical biology, with positive and negative controls included in each run to ensure result reliability.

**Figure 1:**
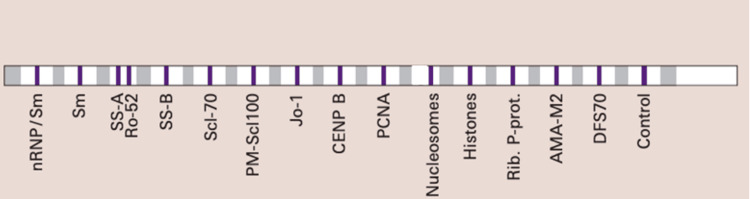
EUROIMMUN immunoblot (ANA Profile 3 Plus DFS-70) ANA: anti-nuclear antibody

Anti-ENA profiling (Sm, RNP/Sm, SSA/Ro52, SSB, Jo-1, Scl-70, nucleosomes, centromeres, Pm-Scl-100, PCNA, histones, AMA-M2, ribosomal P proteins, DFS-70) was carried out using the ANA Profile 3 Plus DFS-70 kit (EUROIMMUN Medizinische Labordiagnostika AG, Lübeck, Germany; Figure 1) and quantified with EUROLineScan (EUROIMMUN Medizinische Labordiagnostika AG, Lübeck, Germany; negative: 0-5; borderline: 6-10; positive: 11-50; strongly positive: >50). The manufacturer's protocols were strictly followed throughout the testing process.

Statistical analysis

Statistical analyses were conducted using Jamovi software version 2.3 (Jamovi, Sydney, Australia) and XLSTAT software version 2023 (Addinsoft, Paris, France). Categorical variables were expressed as frequencies and percentages. Associations between ANA characteristics (pattern and titer) and ENA positivity were assessed using univariate analysis via chi-square (χ²) tests. For binary variables coded as 0 (negative) and 1 (positive), odds ratios (ORs) and 95% confidence intervals (CIs) were calculated based on contingency tables to quantify the strength and direction of the associations. All tests were two-tailed, and statistical significance was set at p<0.05.

## Results

Among the 538 patients, 409 (76%) were women and 129 (24%) were men, with a mean age of 47.7 years. Out of the total number of patients, 326 (60%) tested positive for ANA, including 260 women (48%) and 66 men (12%). ENA antibodies were detected in 268 patients (n=268; 50% of the total cohort): 215 (40%) women and 53 (10%) men. The 40-60 year age group accounted for the most significant proportion of patients in the cohort, with 187 out of 538 individuals (35%). Tables 1-3 summarize the demographic and serological characteristics of the studied population.

**Table 1:** Demographic data of patients

Variables	Values
Average age	47.7
0-20	38 (7%)
20-40	161 (30%)
41-60	187 (35%)
>60	152 (28%)
Females	409 (76%)
Males	129 (24%)

**Table 2:** IIF patterns and titers IIF: immunofluorescence, ANA: anti-nuclear antibody, H-ANA: homogeneous, S-ANA: speckled, C-ANA: cytoplasmic, N-ANA: nucleolar, CE-ANA: centromeric

	Frequency
ANA positive	326 (61%)/538
ANA patterns	
H-ANA	49 (15%)/326
S-ANA	161 (49%)/326
C-ANA	39 (12%) / 326
N-ANA	17 (5%)/ 326
CE-ANA	5 (1%)/ 326
H-ANA + S-ANA	8 (2%)/326
S-ANA + C-ANA	26 (8%)/326
H-ANA + C-ANA	6 (2%)/326
S-ANA + N-ANA	7 (2%)/326
H-ANA + N-ANA	4 (1%)/326
N-ANA + C-ANA	4 (1%)/326
ANA titers	
1/160	174 (53%)/326
1/320	93 (29%) /326
1/640	46 (14%)/326
1/1280	13 (4%)326

**Table 3:** Frequency of positive anti-ENA

Antigenic targets	Frequency
Anti-ENA	268 (49%)/538
Sm	22/268
RNP/Sm	41/268
SS-A	40/268
SS-B	23/268
Ro-52	61/268
Jo-1	22/268
Scl70	23/268
Nucleosomes	21/268
Centromere	16/268
Pm-Scl100	43/268
PCNA	31/268
Histones	31/268
AMA-M2	33/268
Ribosomal protein	14/268
DFS-70	35/268

**Figure 2:**
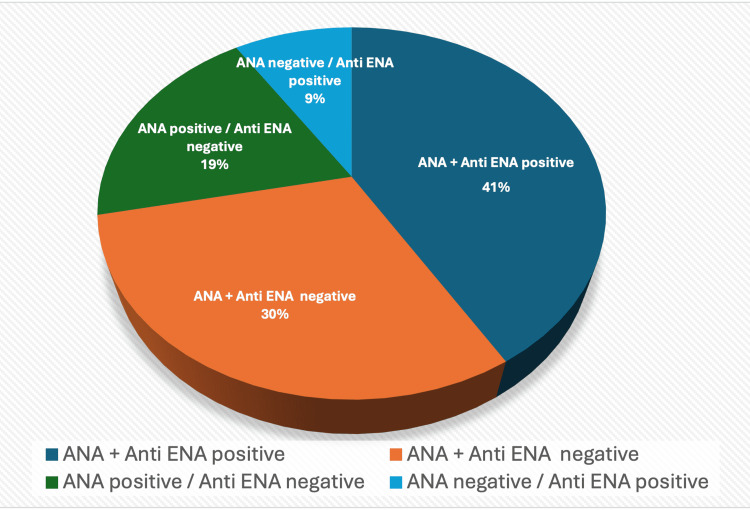
Distribution of patients according to ANA and ENA results ANA: anti-nuclear antibody, ENA: extractable nuclear antigen

A total of 221/538 (41%) patients had positive ANA and ENA results, while 165/538 (30%) were negative for both tests. Additionally, 105/538 (19%) ANA-positive sera tested negative for ENA, whereas 47/538 (9%) ANA-negative sera tested positive for ENA (Figure 2). The most frequently identified IIF patterns among positive patients, both women and men, were 161/326 (49%) S-ANA and 49/326 (15%) H-ANA. Some patients exhibited mixed patterns, most commonly S-ANA + C-ANA in 26/326 patients (8%). We analyzed the most common ENA among different ANA patterns. ENA could be detected at varying frequencies depending on the ANA patterns.

**Figure 3:**
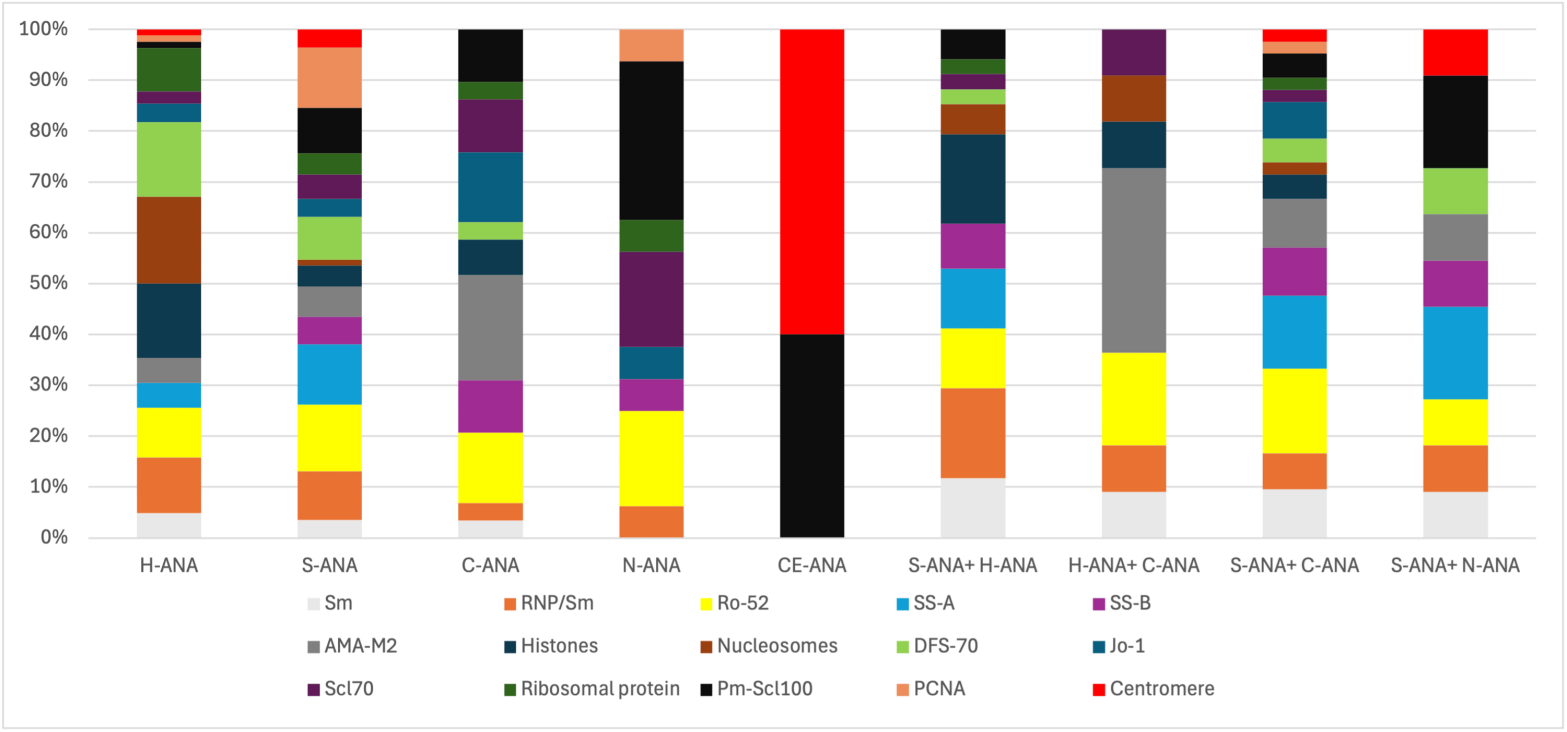
Frequency of ENA in different ANA profiles ENA: extractable nuclear antigen, ANA: anti-nuclear antibody, H-ANA: homogeneous, S-ANA: speckled, C-ANA: cytoplasmic, N-ANA: nucleolar, CE-ANA: centromeric

The most frequently identified ENAs were Ro-52, SS-A, PmScl-100, and Sm/RNP. SS-A/Ro52 (20/268 and 22/268) and PCNA (20/268) were predominant in patients with S-ANA. Nucleosomes (14/268), histones (12/268), and DFS-70 (12/268) were the most frequently found in H-ANA. The AMA-M2 antigen was most frequently seen with cytoplasmic fluorescence (6/268), while Pm-Scl100 (5/268), Scl-70 (3/268), and Ro-52 (3/268) were linked to nucleolar fluorescence. Regarding mixed patterns, the most frequent combination, S-ANA + C-ANA, mainly presented Ro-52 (7/268) and SS-A (6/268) (Figure 3).

Association between ANA patterns and anti-ENA antibodies

We found specific associations between ANA patterns and anti-ENA antibodies. There was an increased frequency of SSA (20/161; p=0.004, OR: 2.75, 95% CI: 1.41-5.35) and PCNA (20/161; p<0.001, OR: 4.78, 95% CI: 2.17-10.5) in patients with speckled patterns. There was an increased frequency of anti-histone antibodies (12/49; p<0.001, OR: 7.85, 95% CI: 3.54-17.4), anti-nucleosome antibodies (14/49; p<0.001, OR: 31.5, 95% CI: 11.4-87.1), anti-DFS 70 antibodies (12/49; p<0.001, OR: 6.43, 95% CI: 2.96-13.9), anti-ribosomal protein antibodies (7/49; p<0.001, OR: 11.2, 95% CI: 3.76-33.6), and anti-Sm/RNP antibodies (9/49; p=0.009, OR: 3.14, 95% CI: 1.4-7.05) in patients with homogeneous fluorescence. We found an increased frequency of anti-Scl70 antibodies (3/17; p=0.034, OR: 5.26, 95% CI: 1.40-19.8) and anti-Pm-scl100 antibodies (5/17; p=0.003, OR: 5.50, 95% CI: 1.84-16.5) in sera with nucleolar fluorescence. The results can be found in Table 4.

**Table 4:** Associations between indirect immunofluorescence patterns and anti-ENA antibodies n/N: number of positive cases over total cases within each ANA pattern group, ENA: extractable nuclear antigen, ANA: anti-nuclear antibody, H-ANA: homogeneous, S-ANA: speckled, C-ANA: cytoplasmic, N-ANA: nucleolar, OR: odds ratio

	Patterns											
ENA	H-ANA (N=49)			S-ANA (N=161)			C-ANA (N=39)			N-ANA (N=17)		
	n/N	p-value	OR	n/N	p-value	OR	n/N	p-Value	OR	n/N	p-value	OR
Sm	4 /49	0.234	2.42 (0.779-7.49)	6/161	1	0.908 (0.246-2.39)	1/39	0.97	0.617 (0.0806-4.72)	0/17	0.824	0.652 (0.037-11.2)
Sm/RNP	9/49	0.009	3.14 (1.4-7.05)	16/161	0.290	1.51 (0.783-2.91)	1/39	0.342	0.295 (0.0395-2.21)	1/17	1	0.736 (0.095-5.69)
SS-A	4/49	1	1.16 (0.395-3.43)	20/161	0.004	2.75 (1.41-5.35)	0/39	0.137	0.148 (0.089-2.46)	0/17	0.476	0.351 (0.0207-5.96)
Ro-52	8/49	0.310	1.67 (0.743-3.77)	22/161	0.249	1.46 (0.826-2.56)	4/39	1	0.921 (0.31-2.69)	3/17	0.618	1.76 (0.495-6.38)
SS-B	0/49	0.247	0.205(0.0123-3.44)	9/161	0.397	1.61 (0.675-3.85)	3/39	0.466	2.06 (0.582-7.3)	1/17	1	1.46 (0.185-11.5)
AMA-M2	4/49	0.643	1.55 (0.517-4.64)	10/161	0.88	1.15 (0.525-2.51)	6/39	0.10	2.73 (0.98-7.58)	0/17	0.62	0.451 (0.0265-7.68)
Histones	12/49	<0.001	7.85 (3.54-17.4)	8/161	0.702	0.782 (0.34-1.79)	2/39	1	0.85 (0.197-3.73)	0/17	0.601	0.436 (0.0256-7.42)
Nucleosomes	14/49	<0.001	31.5 (11.4-87.1)	2/161	0.07	0.244 (0.055-1.06)	0/39	0.394	0.290 (0.0172-4.88)	0/17	0.853	0.685 (0.039-11.8)
DFS-70	12/49	<0.001	6.43 (2.96-13.9)	14/161	0.283	1.57 (0.777-3.17)	1/39	0.468	0.352 (0.0469-2.64)	0/17	0.534	0.384(0.0226-6.51)
Jo-1	3/49	0.67	1.67(0.474-5.8)	6/161	1	0.9 (0.34-2.39)	4/39	0.09	3.17 (1.01-9.94)	1/17	1	1.53 (0.194-12.1)
Scl70	2/49	1	0.928 (0.211-4.08)	8/161	0.82	1.23 (0.510-2.95)	3/39	0.514	1.95 (0.554-6.89)	3/17	0.034	5.26 (1.40-19.8)
Ribosomal protein	7/49	<0.001	11.2 (3.76-33.6)	7/161	0.651	0.614 (0.169-2.23)	1/39	1	0.964 (0.123-7.56)	1/17	0.94	2.39 (0.295-19.4)
Pm-Scl100	1/49	0.196	0.229 (0.0307-1.70)	15/161	0.48	1.35 (0.693-2.62)	3/39	1	0.989(0.291-3.36)	5/17	0.003	5.50 (1.84-16.5)
PCNA	1/49	0.433	0.336 (0.0447-2.52)	20/161	<0.001	4.78 (2.17-10.5)	0/39	0.23	0.198(0.0118-3.30)	0/17	0.1	1.08 (0.138-8.42)
Centromere B	1/49	1	0.692 (0.089-5.38)	6/161	0.698	1.54 (0.539-4.40)	0/39	0.543	0.388 (0.0228-6.60)	0/17	1	0.91 (0.052-15.9)

Association between gender and anti-ENA antibodies

Our analysis revealed significant gender-based differences in anti-ENA antibody profiles. Anti-ENA positivity was substantially higher in women (215 out of 409 women, 53%) than in men (53 out of 129 men, 41%), with this difference reaching statistical significance (p=0.023). Women demonstrated a greater likelihood of anti-ENA positivity (OR: 1.59, 95% CI: 1.06-2.37). The disparity was particularly striking for anti-SSA antibodies: detected in 9% of women (37 out of 409 women) versus only 2% of men (3 out of 129 men). This gender-specific pattern remained statistically significant (p=0.01), with women showing higher odds of anti-SSA positivity (OR: 4.18, 95% CI: 1.27-13.8).

Association between age and anti-ENA antibodies

Anti-ENA antibodies were most frequently detected in the 40-60 age group, where 106 of 187 individuals (57%) tested positive. This association was statistically significant (p=0.02; OR: 1.53, 95% CI: 1.07-2.18).

Association between ANA titer and anti-ENA antibodies

The frequency of anti-ENA antibodies was higher in patients with a moderate (OR: 3.37, 2.22-5.11) and high ANA titer (p<0.001, OR: 28.6, 1.69-483) compared to those with a low titer (p<0.001, OR: 2.16, 95%, 1.49-3.13). Sixty-five of 174 patients (37%) with a low titer did not present anti-ENA antibodies, compared to 40 of 139 (28%) with moderate fluorescence intensity and none of the 13 patients with high fluorescence intensity.

## Discussion

The primary objective of this study was to investigate whether specific ANA patterns and titers, as detected by IIF on HEp-2 cells, are associated with the presence of anti-ENA antibodies in a Moroccan hospital-based population. Detecting ANA is crucial when evaluating systemic autoimmune diseases, particularly when patients exhibit symptoms in multiple organs. But a positive ANA result from standard IIF testing doesn't automatically equal autoimmune disease. These antibodies are also commonly found in non-rheumatic cases [6].

ANA-IIF patterns as predictors of anti-ENA antibody profiles

In this cohort, S-ANA was the most frequently observed ANA profile, noted in 161 of the 326 ANA-positive cases (49%). H-ANA and C-ANA followed in frequency. A notable association was found between S-ANA and anti-SSA antibodies, identified in 20 of the 161 patients (p=0.004; OR: 2.75; 95% CI: 1.41-5.35). This association echoes earlier findings from studies like those of Sanchez-Guerrero et al. (40%) [7], Banhuk et al. (43%) [8], and Lora et al. [9], which all demonstrated a similar connection between S-ANA and the presence of anti-SSA or anti-Ro52 antibodies.

This alignment highlights the clinical significance of S-ANA as a reliable indicator of anti-ENA antibody presence, particularly in cases involving SSA/Ro autoantibodies. It highlights the potential utility of this pattern in directing ENA testing for patients being evaluated for systemic autoimmune conditions.

The association between H-ANA and anti-histone or anti-nucleosome antibodies in our study is consistent with previous findings [10]. Notably, we also observed H-ANA in several patients who were positive for DFS-70, although this antibody typically produces a dense, fine-speckled (DFS) pattern on IIF. Similar discrepancies were reported by Yumuk and Demir [11], who found that nearly half of DFS-70-positive individuals displayed atypical staining patterns. This variability may reflect the coexistence of other autoantibodies, as 10 of 35 DFS-70-positive cases in our study also had anti-histone or anti-nucleosome antibodies. The potential involvement of anti-DNA antibodies, not assessed here, cannot be excluded [12].

H-ANA's association with anti-ribosomal protein antibodies does not align with standard ANA profiles, such as AC-19 (diffuse cytoplasmic fluorescence) or AC-5 (coarse speckled nucleoplasmic fluorescence) for Sm/RNP [13]. Some studies suggest that IIF is not a reliable screening test for anti-RibP antibodies, with detection sensitivity below 30% in certain Hep-2 cell studies [14]. Since anti-Sm/RNP and anti-ribosomal protein antibodies are particular to lupus, their presence might indicate SLE cases with anti-DNA, histone, or nucleosome antibodies contributing to homogeneous fluorescence [15]. In some studies, 28.6% of lupus patients showed an RNP/Sm association with homogeneous fluorescence [16]. In our study, all patients (9/9) with homogeneous fluorescence and positive RNP/Sm ENA were also positive for histones/nucleosomes.

Moreover, N-ANA, although rare, was observed in 17 out of 326 ANA-positive patients (5%) and showed a strong association with anti-Scl70 and anti-Pm-Scl100 antibodies. These findings reinforce the diagnostic value of N-ANA in systemic sclerosis and overlap syndromes, where anti-Scl70 and anti-Pm-Scl100 serve as serological markers for diffuse cutaneous systemic sclerosis and scleroderma-polymyositis overlap, respectively [13].

However, it is important to note that ANA positivity is not exclusive to autoimmune diseases; it may also occur in viral infections, drug-induced autoimmune reactions, or in healthy elderly individuals, especially at low titers [2].

High ANA titers strongly correlate with ENA positivity

ANA titers of 1:320 or higher (1:640, ≥1:1280) are generally observed in patients with autoimmune diseases. In contrast, low-titer ANA (such as 1:160) may be seen in both autoimmune conditions and occasionally in healthy individuals. Our findings align with studies showing that samples with high fluorescence intensity at a fixed titer have an increased frequency of anti-ENA antibodies [17].

Gender-specific differences in anti-ENA antibody prevalence

Among the 40 SSA-positive sera, 37 were from women and only three from men. A strong association was found between anti-SSA and female sex (OR: 4.18), consistent with previous studies, especially in Sjögren's syndrome, where anti-SSA/Ro is predominant in women, reaching 94% in some studies [6]. Estrogen and other sex hormones may contribute to a higher prevalence of autoimmunity and increased ANA positivity in women, with levels rising with age [18,19]. However, the high proportion of women in our study, 409 out of 538 patients (76%), may have influenced these findings.

The study has several strengths. First, a large number of patients (n=538) were included in the study, which contributes to making the statistical analysis more robust and provides insight into ANA/ENA testing in the Moroccan population. Second, we analyzed several parameters, including age, sex, ANA titer, and IIF patterns, highlighting their associations with anti-ENA. In addition, using a 15-antigen immunoblot panel for ENA testing, more than several studies on the same subject enabled us to conduct a more precise analysis of the effect of different variables on ENA positivity. Finally, our observations were reinforced by the fact that patients with autoimmune diseases were excluded from the study, which allowed us to reduce the potential confounding effect of immunosuppressive treatments on anti-nuclear antibody levels.

Three methodological constraints deserve attention. The retrospective, single-center design may introduce referral bias and limit the ability to draw causal conclusions. Lack of comprehensive clinical data (particularly symptoms and diagnoses) also restricts serological interpretation. Future studies require covariate adjustment to establish the true predictive utility of ANA across populations. Multivariate analysis would clarify the interactions between key variables, such as titer levels and fluorescence patterns. Although EUROIMMUN kits demonstrated consistent performance, unverified cases obtained through methods such as ELISA can affect the reliability of certain findings. Crucially, since all participants were referred explicitly for ANA testing, our results may not extend to general screening contexts.

Additionally, the observed discordance between ANA-negative and ENA-positive results in 47 of 538 patients (9% of cases) may reflect differences in assay sensitivity or antigen coverage between IIF and immunoblot techniques [20]. Finally, the presence of ENA negativity in 105 out of 538 ANA-positive patients (19%) highlights the limited specificity of IIF, as ANA positivity may also be observed in non-autoimmune conditions or even in healthy individuals [17].

## Conclusions

Our data clearly show that high ANA titers and specific patterns, particularly S-ANA and H-ANA, reliably indicate the presence of anti-ENA antibodies, confirming their diagnostic value in autoimmune workups. C-ANA, in contrast, proved less clinically useful. The increased ENA positivity we noted in women and those aged 40-60 years likely reflects referral patterns in our cohort rather than true biological trends. These observations highlight a critical need: refining how labs interpret ANA profiles to target ENA testing smartly. While lab results provide key pieces of the puzzle, they're just one part. We always combine ANA findings with the patient's comprehensive clinical picture, including symptoms, examination findings, and suspected diagnoses, to reach accurate conclusions.
